# Every Man His Own Tailor

**Published:** 1891-05

**Authors:** 


### EVERY MAN HIS OWN TAILOR.

The national costume of the people of India has been much praised for its simplicity, lightness and adaptability to the climate of the country. The saree, the dhotur and the turban are capable of being manufactured in various tints and colors, and of being folded and displayed on the person in various ways. The turban possesses the greatest adaptability to the taste of individuals, and we find that this taste has been exercised by the people to distinguish the sect of the wearer, and in some cases the priests. But the ingenuity that has been exercised in the form and color of the chief articles of dress of the people of this country is not the ingenuity of the tailor but the ingenuity of a people happily ignorant of the tailor's art. They are worn by the people exactly as they pass from the weaving-looms; hence when presents of cloth are made in families—and the custom of making such presents is general—these presents are described as "cloths." A bride and bridegroom receiving a present of cloth at a wedding ceremony are at once dressed in complete suits of "cloths." Sarees, dhoturs and turbans are simply cloths of various lengths, especially the turbans; and it is not at all necessary that the wearer of the cloth and the cloth itself should be of any relative size, for these cloths fits any body or any body fits the garment. The chief idea which appears to run through the Indian national costume is how to make nature do all the tailoring. Tailor-made made clothing has been introduced into India since the importation of needles and thread ; but the saree, though made brighter by gay colors than formerly, still retains its distinction as a garment that requires no tailoring to fit it to the female form. Throughout the villages of India soap is regarded as a natural curiosity, and is never kept in stock by the village shopkeeper. It is, however, finding a place in the large towns in the shops of grocery dealers, who do a retail business in eau-de-cologne, but the consumption is by no means considerable. The total consumption of soap in this country does not exceed 100,000 hundred-weight per annum, or one hundred-weight among 2,500 persons.—*Times of India.*

